# Association mapping of North American spring wheat breeding germplasm reveals loci conferring resistance to Ug99 and other African stem rust races

**DOI:** 10.1186/s12870-015-0628-9

**Published:** 2015-10-14

**Authors:** P. Bajgain, MN Rouse, P. Bulli, S. Bhavani, T. Gordon, R. Wanyera, PN Njau, W. Legesse, JA Anderson, MO Pumphrey

**Affiliations:** Department of Agronomy, Purdue University, 915 West State Street, West Lafayette, IN 47907 USA; Department of Agronomy and Plant Genetics, University of Minnesota, St. Paul, MN 55108 USA; United States Department of Agriculture-Agricultural Research Service (USDA-ARS), Cereal Disease Laboratory, St. Paul, MN 55108 USA; Department of Plant Pathology, University of Minnesota, St. Paul, MN 55108 USA; Department of Crop and Soil Sciences, Washington State University, Pullman, WA 99164 USA; International Maize and Wheat Improvement Center (CIMMYT), ICRAF House, United Nations Avenue, Gigiri, Nairobi, Kenya; United States Department of Agriculture-Agricultural Research Service (USDA-ARS), Aberdeen, ID 83210 USA; Kenya Agricultural and Livestock Research Organization (KALRO), Njoro, Kenya; Ethiopian Institute of Agricultural Research (EIAR), Pawe, Ethiopia

**Keywords:** Genome-wide association study, Stem rust of wheat, Ug99, Adult plant resistance, All-stage resistance, Resistance breeding

## Abstract

**Background:**

The recently identified *Puccinia graminis* f. sp. *tritici* (*Pgt*) race TTKSK (Ug99) poses a severe threat to global wheat production because of its broad virulence on several widely deployed resistance genes. Additional virulences have been detected in the Ug99 group of races, and the spread of this race group has been documented across wheat growing regions in Africa, the Middle East (Yemen), and West Asia (Iran). Other broadly virulent *Pgt* races, such as TRTTF and TKTTF, present further difficulties in maintaining abundant genetic resistance for their effective use in wheat breeding against this destructive fungal disease of wheat. In an effort to identify loci conferring resistance to these races, a genome-wide association study was carried out on a panel of 250 spring wheat breeding lines from the International Maize and Wheat Improvement Center (CIMMYT), six wheat breeding programs in the United States and three wheat breeding programs in Canada.

**Results:**

The lines included in this study were grouped into two major clusters, based on the results of principal component analysis using 23,976 SNP markers. Upon screening for adult plant resistance (APR) to Ug99 during 2013 and 2014 in artificial stem rust screening nurseries at Njoro, Kenya and at Debre Zeit, Ethiopia, several wheat lines were found to exhibit APR. The lines were also screened for resistance at the seedling stage against races TTKSK, TRTTF, and TKTTF at USDA-ARS Cereal Disease Laboratory in St. Paul, Minnesota; and only 9 of the 250 lines displayed seedling resistance to all the races. Using a mixed linear model, 27 SNP markers associated with APR against Ug99 were detected, including markers linked with the known APR gene *Sr2*. Using the same model, 23, 86, and 111 SNP markers associated with seedling resistance against races TTKSK, TRTTF, and TKTTF were identified, respectively. These included markers linked to the genes *Sr8a* and *Sr11* providing seedling resistance to races TRTTF and TKTTF, respectively. We also identified putatively novel *Sr* resistance genes on chromosomes 3B, 4D, 5A, 5B, 6A, 7A, and 7B.

**Conclusion:**

Our results demonstrate that the North American wheat breeding lines have several resistance loci that provide APR and seedling resistance to highly virulent *Pgt* races. Using the resistant lines and the SNP markers identified in this study, marker-assisted resistance breeding can assist in development of varieties with elevated levels of resistance to virulent stem rust races including TTKSK.

**Electronic supplementary material:**

The online version of this article (doi:10.1186/s12870-015-0628-9) contains supplementary material, which is available to authorized users.

## Background

Stem rust of wheat, caused by the fungal pathogen *Puccinia graminis* Pers. f. sp. *tritici* (*Pgt*), is considered potentially the most damaging disease of wheat. Historical crop losses caused by stem rust epidemics have been recorded in all wheat growing regions of the world [1, 2]. This disease has been primarily controlled via genetic resistance with resistance genes discovered in bread wheat and its relative species [3–5]. However, the emergence of the highly virulent stem rust race TTKSK (also known as Ug99) and its variants has rendered many of the resistance genes ineffective to the pathogen [5, 6], and threatens global wheat production and supply.

The race TTKSK [7], first observed in Uganda in 1998 was found to be virulent to *Sr31* [8], a widely deployed and important stem rust resistance gene. Within a few years, virulence to three other resistance genes was documented in the Ug99 lineage: *Sr24* was defeated by race TTKST [7], *Sr36* was defeated by race TTTSK [7, 9], and *Sr9h* was defeated by race TTKSF+ [10, 11]. The urediniospores of the stem rust fungus can travel long distances with the flow of wind [1, 12]. Consequently, Ug99 race groups have traveled from hotspot areas in East Africa to South Africa and Iran in the North [13, 14]. The Ug99 race group is projected to spread further to other wheat growing areas in the world [5, 14, 15].

Realizing the threat posed by the Ug99 race group, over 200,000 wheat lines including accessions from germplasm collections to breeding materials from wheat breeding programs throughout the world were screened for resistance to Ug99 in Kenya and Ethiopia [5]. The results showed that 85-95 % of wheat lines grown globally are susceptible to Ug99. The results obtained from screening global germplasm highlights the risk looming over worldwide wheat production due to the susceptibility of current varieties. It is therefore essential that resistance genes are identified and used in breeding programs in these areas, including North America, to prepare for the possible arrival of the Ug99 race group and other highly virulent races. One of such highly virulent races is the newly identified race TKTTF, which has a different genetic lineage from the Ug99 race group [16]. This race caused yield losses close to 100 % on the most widely grown wheat cultivar, ’Digalu’, in southeastern regions of Ethiopia in 2013–2014 [16]. Hence, there is urgent need to identify and characterize new genes for resistance to such races, and their rapid incorporation in the breeding pipeline to develop varieties with improved level of resistance.

Two main types of resistance strategies are used in wheat breeding against stem rust: 1) all-stage resistance (ASR) or seedling resistance, and 2) adult plant resistance (APR). ASR is usually characterized by a hypersensitive reaction upon fungal attack, and usually confers a high level of resistance that is effective in all stages of plant development [17]. On the contrary, APR is generally expressed during the adult growth stages of the plant, usually beginning at booting stage. The major drawback of ASR is the high likelihood of the gene being defeated by new pathogen races when resistance genes are deployed singly, in so called “boom and bust cycles” [18]. While APR genes are considered more durable to wheat rusts than ASR genes, they may not provide adequate levels of resistance to high disease pressure [19]. Therefore, a gene-pyramiding strategy that utilizes a few seedling genes or 4–5 APR genes or genes of both resistance types would be highly desirable. Discovery and development of reliable markers for effective marker-assisted gene introgression and selection is vital to routinely combine resistance genes. Such genes and linked markers can be identified in primary, secondary, and tertiary gene pools of wheat and its related species. However, to minimize linkage drag during resistance gene introgression from wild relatives and increase breeding efficiency, discovery of resistant material in existing breeding programs is preferable.

Association mapping (AM), or linkage disequilibrium (LD) mapping, is a powerful technique used to identify marker-trait associations, and has been used successfully in several crop species such as wheat, barley, soybean, and maize [20–22]. The AM strategy exploits historical recombination events existing in the lines being studied, which can range from natural collections to breeding populations. Since existing populations can be used for mapping loci associated with traits of interest, as opposed to specifically designed populations, this approach is widely used to study the genetic makeup controlling trait variation in wider germplasm pools [22, 23]. As allelic variation and marker polymorphisms are observed at a higher frequency in a genome-wide association study (GWAS) panel compared to a biparental population [24, 25], useful and novel alleles associated with traits of interest may be identified when pairing with high-throughput marker technologies. One drawback of AM is that the underlying population stratification due to breeding history, selection, genetic drift, or founder effects can lead to false associations [26, 27]. This issue, however, can be reduced by accounting for population structure using the relationship matrix or distance matrix among the lines [28].

The objective of this study was to evaluate spring wheat lines from nine breeding programs in the United States and Canada, in addition to CIMMYT lines, for their field-based resistance to the Ug99 race group and conduct a GWAS to identify loci associated with resistance. We also made an attempt to distinguish between loci associated with APR and seedling resistance to exotic virulent *Pgt* races TTKSK, TRTTF, and TKTTF.

## Methods

### Plant material

Two-hundred and fifty spring wheat lines from wheat breeding programs in North America were assembled as part of the Triticeae Coordinated Agricultural Project (www.triticeaecap.org). Elite lines were representative of the following wheat breeding programs in the United States: Montana State University (MSU), South Dakota State University (SDSU), University of California-Davis (UCD), University of Idaho (UI), University of Minnesota (UMN), and Washington State University (WSU); in Canada: Agriculture and Agri-Food Canada (Ag-Canada) Manitoba, Ag-Canada Saskatchewan, and Ag-Canada Alberta; and in Mexico: the International Maize and Wheat Improvement Center (CIMMYT). This germplasm panel was previously used to assess root morphology traits [29].

### Field stem rust evaluation

A panel of 250 lines was evaluated for field response to stem rust in four disease environments: at Njoro, Kenya during the off-season (January to April 2013), and the main-season (June to October 2013), and at Debre Zeit, Ethiopia during the off-season (January to June 2013, and 2014). These environments are referred in the text as KenOff13, KenMain13, EthOff13, and EthOff14, respectively. In all environments, phenotypic data was collected from plant heading stage up to grain maturing stage (i.e. between Zadoks 50 and 90) [30], when the susceptible checks reached maximum severity (usually ~80 % severity).

In Njoro, plots were arranged in an augmented design with the lines represented once and the susceptible check line ‘Red Bobs’ planted after every fifty entries. The lines were sown as 70 cm long twin rows, 20 cm apart, flat bed. Spreader rows were sown perpendicular to the twin rows, surrounding the field to initiate disease development and maintain uniform disease pressure in the nursery. The spreader rows comprised of a mixture of lines susceptible to race TTKST (Ug99 + *Sr24* virulence): ‘CCK’ (Canadian Cunningham Kennedy), ‘PBW343’, ‘Morocco’ and few susceptible CIMMYT lines. The disease was initiated by inoculating the spreader rows using a bulk inoculum of *Pgt* urediniospores collected at the Njoro field site. Wheat stem rust differential lines with known stem rust resistance genes indicated that the predominant, if not only, race present in the nursery since 2008 was race TTKST; [31]). The urediniospores were suspended in water and injected into spreader plants at 1 m distance prior to booting (growth stage Z35-Z37; [30]). The spreader plants were then sprayed with urediniospores suspended in light mineral oil Soltrol 170 (Chevron Phillips Chemical Company, The Woodlands, TX).

The nursery in Debre Zeit was set up similar to the Njoro nursery. Lines were planted in 1 m long twin rows, flanked by spreader rows comprised of a mixture of susceptible wheat varieties ‘PBW343’, ‘Morocco’, and ‘Local Red’. The spreader rows were artificially inoculated with a bulk of fresh urediniospores collected from PBW343 (PBW343 has *Sr31* and several races in the Ug99 race group are virulent to *Sr31*) and also collected from local fields. Inoculation was carried out in Debre Zeit as it was in the Njoro nursery.

Disease severity on a 0-100 % modified Cobb scale [32] and infection response [17] were recorded for each line. Severity and infection response notes were recorded 2–3 times during the season; the terminal data, which exhibited better disease segregation among the lines in the panel, was used in the analysis. The infection response to disease was assigned constant values as recommended by Stubbs *et al*. [33] with the response types ‘resistant – moderately resistant’ and ‘moderately susceptible – susceptible’ coded as 0.3 and 0.9, respectively. The stem rust severity values were multiplied by the infection response values to obtain coefficient of infection values [33], which were used in subsequent analyses.

### Seedling stem rust evaluation

Seedling assays were conducted at the United States Department of Agriculture, Agricultural Research Service (USDA-ARS) Cereal Disease Laboratory during the winter months between December and February starting December 2012 through February 2014. The 250 spring wheat lines were evaluated with three virulent races of *Pgt* race TTKSK (isolate 04KEN156/04, Ug99), race TRTTF (isolate 06YEM34-1), and race TKTTF (isolate 13ETH18-1). Race TRTTF was detected in Yemen and Ethiopia and characterized as broadly virulent to wheat stem rust resistance genes including *Sr13* and *Sr1RS*^*Amigo*^ [34]. Seedling assays were performed as described previously by Rouse *et al*. [35] for evaluating wheat germplasm for reaction to *Pgt* races. Two biological replicates of the seedling assays were performed for each *Pgt* race. Infection types (ITs) were recorded on a 0 to 4 scale according to Stakman *et al*. [36]. ITs less than or equal to 2+ are considered low infection types whereas ITs greater than or equal to 3- are considered high infection types [36]. In order to use the Stakman ITs in the GWAS, the 0–4 scale was converted to a 0–9 linear scale as proposed by Zhang *et al*. [37] (Additional file 1). The average linear scale score across the two replications was used in the AM analyses.

### Statistical analysis

Data sets from each field environment were fitted into a mixed model with environment as a fixed effect and wheat lines as a random effect to correct for data distortion due to trial effects. Using this model, best linear unbiased predictors (BLUPs) for each line were predicted from the combined analysis model using SAS 9.1, from which final corrected trait values were obtained. After the mean values were normalized for each environment, trait values for each line in all environments were averaged, and used for genome-wide mapping. Heritability on an entry mean basis was calculated based on the method described by Holland *et al*. [38].

### SNP genotyping and analysis of molecular data

The AM panel was genotyped at the USDA-ARS Cereal Crops Research Unit, Fargo, ND as part of the TCAP project for 90,000 gene-based SNPs using a custom Infinium iSelect bead chip assay following the manufacturer’s instructions (Illumina Inc., Hayward, CA) [39]. Allele calls were performed using the computer program GenomeStudio v2011.1 (Illumina Inc.). *As genotyping of these lines was carried out as a part of the TCAP, the genotype data was obtained from The Triticeae Toolbox. During data download,* SNP markers with minor allele frequency less than 0.05 and no calls for more than 10 % of the lines were removed from the dataset. Lines that were genotyped at less than 20 % loci were also removed from the dataset. These filtering steps yielded 23,976 SNP markers and 241 lines that were used for genome-wide association analysis.

Principal component analysis (PCA) on the marker data of the lines was performed by using the Genomic Association and Prediction Integrated Tool (GAPIT) R package [40]. For the purpose of displaying the PCA results, the R package ‘princomp’ was used to reconstruct the covariance matrix. Principal component 1 scores were plotted against principal component 2 scores for each line in the mapping panel. In order to confirm the results of PCA analysis, the lines were also clustered by their genetic distances using JMP Pro 11.0.0 (SAS Institute Inc.) with Ward’s hierarchical clustering method [41].

Linkage disequilibrium between the markers was estimated as squared allele-frequency correlations (r^2^). The package ‘LDheatmap’ in R was used to calculate r^2^ in each of the A, B, and D genomes of common wheat. The LD decay between the marker pairs for each genome was estimated using the least squares regression function, and is represented by the exponential curve. Map positions of the SNPs were obtained from Wang *et al*. [39].

### Linkage disequilibrium and association analysis

A total of 18,302 mapped SNP markers were used to estimate pairwise LD. LD between markers were calculated for the A, B, and D genomes, and plotted against the genetic distance in centimorgans (cM). The extent of LD between marker pairs was visualized by fitting locally weighted polynomial regression (LOESS) curves into the scatter plot.

Genome-wide association analysis investigating the marker-trait association was performed using the R package GAPIT [40], with the growth stages of genotypes included as covariate for *Pgt* resistance. The population parameters previously determined (P3D) model [42] was used to conduct association analyses with all trait data. Based on the model selection using the Bayesian information content criterion (BIC), a kinship-mixed linear model (K-MLM) approach that accounts for Type I error rate due to relatedness was used for all traits.

## Results

### Phenotypic data

**Fig. 1:**
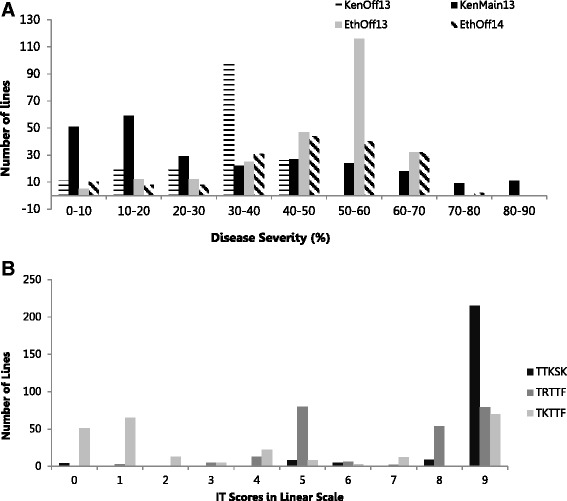
Frequency distribution of stem rust severity scores for the North American Spring Wheat Breeding Germplasm (250 lines) during (**a**) four seasons in East Africa; (**b**) screening for resistance at the seedling stage with three stem rust races. Seedling infection type (IT) scores have been converted to the linear scale, as discussed in ‘Methods’

Adequate disease pressure was observed in each environment to discriminate among the entries, as indicated by highly significant (*p* < 0.01) F-values from ANOVA results of the field data (results not shown). Distribution of rust severities in all environments indicated a quantitative mode of disease distribution with the Njoro main season (KenMain13) recording both higher severity and wider distribution of scores (Fig. 1a). On average however, disease severity was relatively lower in Njoro environments with mean values of 33 % during the 2013 offseason and 35 % during the 2013 main season, compared to Debre Zeit, Ethiopia with 52 % severity during the 2013 offseason and 50 % during the 2014 offseason. Moderate correlations (*r* ranging from 0.44 to 0.57) between the field data from the four seasons was observed (Table 1). The estimated broad sense heritability across the four environments was 0.72.

**Table 1:** Pearson correlation coefficients for stem rust severity observed in four field environments

	EthOff13	EthOff14	KenOff13
EthOff13^a^			
EthOff14^b^	0.52		
KenOff13^c^	0.57	0.44	
KenMain13^d^	0.46	0.46	0.47

All correlation coefficient values are significant at *p* < 0.001

^a^2013 off-season (January to June) disease nursery in Debre Zeit, Ethiopia

^b^2014 off-season disease nursery in Debre Zeit, Ethiopia

^c^2013 off-season (January to April) disease nursery in Njoro, Kenya

^d^2013 main-season (June to October) disease nursery in Njoro, Kenya

The complete panel of 250 lines was inoculated with *Pgt* races TTKSK, TRTTF, and TKTTF at seedlings to uncover genetic factors contributing resistance to these virulent stem rust races. Replications of the seedling tests were highly correlated with r-value ranging from 0.84-0.99 (*p* < 0.001). However, the pair-wise correlations among the three races were low and not significant (data not shown). Most of the lines screened for seedling resistance against race TTKSK were susceptible, and only 15 (6 %) showed resistant reactions (IT 22+ or lower) (Fig. 1b). Seedling resistance was more common to races TRTTF, with 44 %, and TKTTF, with 63 % of lines with low infection types. Of the seedling resistant lines, nine lines showed resistance to all three races; three lines showed resistance only to races TTKSK and TRTTF; and five lines showed resistance only to races TTKSK and TKTTF (Additional file 1). The University of Minnesota cultivar ‘Thatcher’ was heterogeneous for resistance to race TTKSK (IT 0; / X in the first replication and IT 0; / 3+ in the second replication) yet susceptible to both TRTTF and TKTTF (IT 33+ to both races in both replications). The average adult plant disease severity of lines with seedling resistance to race TTKSK was lower in all four environments than lines susceptible to this race (t-test *p*-value of 0.02 at α = 0.05; Additional file 1).

### Population structure

**Fig. 2:**
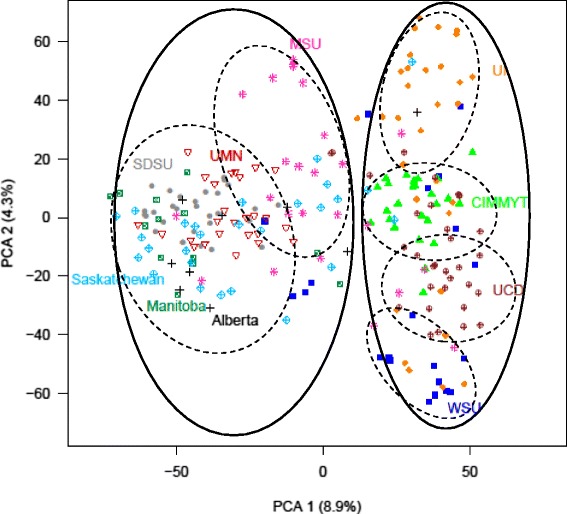
A scatter plot of principal component 1 (PC1) plotted against principal component 2 (PC2). All 241 lines in the AM panel are represented by a symbol, with lines of each breeding program labeled by different symbol and distinct color. Proportion of the variance explained by the principal component values are indicated in parenthesis

To investigate the population structure of the germplasm panel, the genotypes were analyzed for clustering based on principal component (PC) values. The groupings of lines belonging to each breeding program based on PC values are presented in Fig. 2. The first and second PC values explained 8.9 % and 4.3 % genetic variation in the panel, respectively. Although the genetic relationship among representative lines of different breeding programs was not very distinct, a general statement can be made that two main clusters are observed based on genetic differentiation. The cluster on the left of Fig. 1 (hereafter referred to as Cluster 1) comprises lines from breeding programs in the Upper-Midwest of the United States: MSU, SDSU, and UMN; and in Canada. The cluster on the right (hereafter referred to as Cluster 2) comprises lines from the Western United States: UCD, UI, and WSU; and in Mexico: CIMMYT. The CIMMYT lines included in this analysis were selected because of their potential heat and drought stress resistance and may not be representative of the entire CIMMYT breeding germplasm. Despite the noticeable amount of relatedness among breeding programs, these two main clusters may be divided further into six sub-clusters: Cluster 1 into two sub-clusters with lines from MSU clustering separately while lines from Alberta, Manitoba, Saskatchewan, SDSU, and UMN form a single sub-cluster; and Cluster 2 into four sub-clusters with lines from CIMMYT, UCD, UI, and WSU. The population stratification and germplasm sharing among the lines revealed by the PC was also corroborated by the results from hierarchical clustering using Ward’s method in JMP (Additional file 1).

### Linkage disequilibrium

**Fig. 3:**
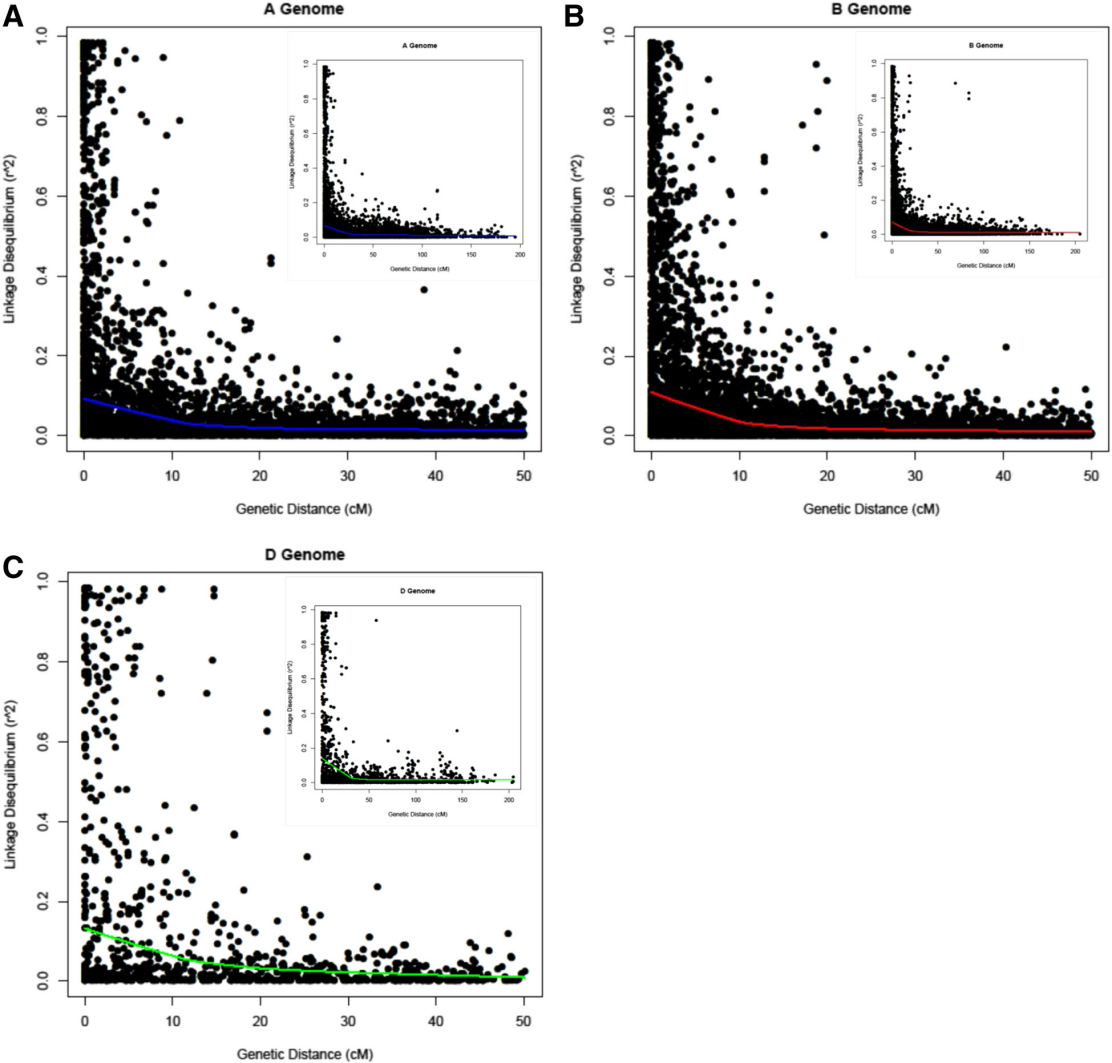
A scatter plot showing squared allele-frequency correlations (r^2^) distribution plotted against genetic distance (cM) for each subgenome of wheat: Figs. 3**a**, **b**, and **c** represent the A, B, and D subgenomes of wheat, respectively. The decline of linkage disequilibrium (LD) is shown by fitting a locally weighed polynomial regression (LOESS) curve into the plot. The inset shows a zoomed-out view of the subgenome whereas the main picture shows a zoomed-in view of the distribution within 50 cM distance

In the A-genome, LD declined to 50 % of its original value at about 8 cM (Fig. 3a), whereas these values for the B-genome and the D-genome were about 7 cM and 6 cM, respectively (Figs. 3b and c, respectively). These values are similar to the LD values reported by Chao *et al.* [43] in their detailed LD characterization of wheat varieties having different growth habits from several breeding programs.

### Association analysis

#### APR mapping

Initial association analysis was conducted on all 241 lines, without removing any lines that showed resistance to race TTKSK during seedling screening of the lines as described later. This approach detected 24 SNP markers on seven different chromosomes (2A, 2B, 3B, 4A, 6A, 6B, and 7A) that were significantly associated (*p*-value <0.001) with field resistance to Ug99 (Table 2). In addition, five significant markers with unknown map positions were detected. The phenotypic variance explained by these 29 SNP markers ranged from 0.2 % to 4.6 %.

**Table 2:** List of SNPs significantly associated with APR to the Ug99 race group in Kenya and Ethiopia. ‘Chromosome 0’ includes all unmapped SNP markers

SNP^a^	Chr^b^	Pos (cM)	*P* value	R^2^ (%)^c^	KenOff13^d^	KenMain13^e^	EthOff13^f^	EthOff14^g^
IWB1351	0	0.0	9.18E-04	1.4	-	-	-	+
**IWB11987**			2.71E-04	4.0	-	-	-	-
IWB13304			9.18E-04	1.4	-	-	-	+
IWB20617			8.92E-04	0.5	-	-	-	-
IWB40153			9.18E-04	1.4	-	-	-	+
**IWB65634**			2.18E-04	3.9	-	-	-	-
IWA3120	1B	90.3	9.07E-04	0.6	-	-	-	-
IWB21176			9.64E-04	0.6	-	-	+	-
IWB31027			9.64E-04	0.6	-	-	+	-
IWB56771			9.34E-04	0.6	-	-	+	-
IWB59663			9.64E-04	0.6	-	-	+	-
IWB49915	2A	122.5	9.18E-04	1.4	-	-	-	+
IWB49914		123.6	9.18E-04	1.4	-	-	-	+
IWB22672		159.7	9.98E-04	0.6	-	-	+	-
IWB2369	2B	48.5	7.68E-04	0.4	-	-	-	-
IWA4275		105.9	8.75E-04	1.4	-	-	-	-
IWA8534		126.1	1.80E-04	0.3	-	+	-	-
IWB23660		126.3	1.79E-04	0.3	-	+	-	-
IWB25868			3.11E-04	0.7	-	+	-	-
IWB69631			3.11E-04	0.7	-	+	-	-
IWB25869		126.5	3.11E-04	0.7	-	+	-	-
IWB32143		157.2	5.05E-04	4.6	-	-	-	-
SNP^a^	Chr^b^	Pos (cM)	P value	R^2^ (%)^c^	KenOff13^d^	KenMain13^e^	EthOff13^f^	EthOff14^g^
IWB8481	2D	9.2	8.44E-04	0.5	-	-	-	-
IWA5203	3B	11.5	7.97E-04	0.4	-	-	-	-
IWB30730			7.74E-04	1.3	-	-	-	-
**IWB12193**		11.6	3.24E-04	4.2	-	+	-	-
IWB49924			5.65E-04	0.2	-	-	-	-
IWB65737			9.15E-04	0.6	-	-	-	-
IWB60424		13.8	5.02E-04	0.1	-	-	-	-
IWB36021		14.1	8.31E-04	1.3	-	-	-	-
IWA2493		32.2	1.49E-04	0.2	-	-	+	-
IWB40004	4A	30.9	9.12E-04	0.6	-	+	-	-
**IWB52694**		43.4	9.96E-05	3.2	-	+	-	-
IWB46973		47.0	8.26E-04	1.3	-	+	-	-
IWB56556			7.54E-04	1.3	-	-	-	-
IWB67877			8.34E-04	1.3	-	-	-	-
IWB47184	5A	69.6	3.44E-04	0.3	-	-	+	-
IWA233	6A	66.0	8.48E-04	1.3	-	+	-	-
IWB24757	6B	119.7	6.33E-04	1.1	-	-	-	-
IWB35697			1.77E-05	1.3	-	-	+	+
IWB6474			6.33E-04	1.1	-	-	-	-
IWB45581		120.6	3.00E-04	0.6	-	-	-	-
**IWB5070**	7A	211.0	2.19E-04	3.9	-	-	-	-
IWB1874		212.7	3.18E-04	0.3	-	-	-	-
**IWB4830**			4.50E-04	4.5	-	-	+	-
IWB62560		213.2	2.30E-04	0.6	-	-	-	-
IWB47548	7B	153.4	6.50E-04	0.3	-	-	-	-
IWA4175		177.1	9.52E-04	0.6	-	-	-	-

^a^Underlined SNP markers were detected during the ‘combined’ mapping approach but not during the ‘APR-specific’ mapping approach. SNP markers in bold were detected in both mapping approaches

^b^Chr ‘0’ indicates unmapped SNPs that were significant in the analysis

^c^R^2^Indicates percent of phenotypic variation explained by the significant locus

^d-g^The ‘+’ sign indicates that the SNP was also detected in GWAS results in each of the environments. The ‘-‘ sign indicates that the SNP was detected only in combined analysis of all environments, and not in individual environments

As the germplasm in the panel was known to possess ASR genes that are effective to the Ug99 race group, it was assumed that these ASR genes could lead to the masking of potential APR genes. Therefore, in an attempt to detect loci conferring APR, lines that were resistant to race TTKSK during seedling screening were removed. Lines that were resistant (IT ranging from 0 to 2+) in either of the two replications of seedling screening, and with a complex score with low and high ITs (for example 2 + 3-) were also removed. This filtering step yielded a subset of 219 lines for the APR-specific genome-wide association analysis.

The APR-specific AM approach identified 26 SNP markers providing APR to Ug99 race group (Table 2). Of the 26 significant SNPs, 23 SNPs were distributed across nine chromosomes (1B, 2A, 2B, 2D, 3B, 4A, 5A, 7A, 7B), whereas the remaining three SNPs were unmapped. Six of the 23 mapped SNPs were also detected in the initial analysis on the whole set of 241 lines. The R^2^ values for the significant SNPs ranged from 0.1 % to 0.6 % (Table 2).

Significant SNP markers detected on the complete panel and by APR-specific mapping were cross-checked with the GWAS results for each of the four environments. While no significant SNPs were detected in the Kenya 2013 offseason environment, three, six, and nine SNPs were confirmed in the Ethiopia offseason 2013, Ethiopia offseason 2014, and Kenya main-season 2014, respectively (Table 2). Two SNPs – *IWA3120* (mapped to 1B) and *IWB35697* (mapped to 6B) were common between the Ethiopia 2013 and 2014 environments. No SNP markers were common in all four environments.

### Mapping of seedling resistance

The genome-wide scan for SNPs linked with seedling resistance to race TTKSK detected 16 significant SNP markers on chromosomes 1D, 3B, 4A, and 5B, and 7 additional significant SNP markers with no mapped locations (Table 3). These SNPs explained 2 to 7 % of variation observed in race TTKSK seedling resistance. The results also revealed that the loci conferring seedling resistance to TTKSK are different than those involved in APR to Ug99 (Table 3, Table 2).

**Table 3:** List of SNPs significantly associated with seedling resistance to Ug99 (race TTKSK)

SNP	Chr^a^	Pos (cM)	*P* value	R^2^ (%)^b^
IWB2159	0	0.0	3.15E-06	4.8
IWB14222			3.45E-04	2.8
IWB36324			6.44E-04	2.5
IWB37066			3.62E-06	4.8
IWB40312			1.97E-07	6.1
IWB49537			2.81E-04	2.9
IWB68926			3.96E-05	3.7
IWA642	1D	67.7	1.32E-04	3.2
IWB24497	3B	67.5	8.43E-06	4.4
IWB30621			1.97E-07	6.1
IWB42046			8.27E-08	6.5
IWB4823			8.43E-06	4.4
IWB56471			1.97E-07	6.1
IWB61425			8.43E-06	4.4
IWB59929		74.4	3.91E-05	3.7
IWB9451		76.9	3.36E-04	2.8
IWA5363	4A	40.3	7.90E-04	2.4
IWA3394	5B	132.3	2.29E-05	3.9
IWB7593			6.14E-04	2.5
IWB822		134.1	5.30E-04	2.6
IWB46318		215.7	1.56E-05	4.1
IWA2099		216.7	9.72E-05	3.3
IWA2100			1.13E-04	3.3

^a^Chr ‘0’ indicates unmapped SNPs that were significant in the analysis

^b^R^2^Indicates the percent of phenotypic variation explained by the significant locus

Similarly, GWAS was conducted for resistance to race TRTTF, a virulent race of Yemeni origin. This resulted in detection of 77 significant SNPs on six chromosomes (1B, 1D, 5D, 6A, 6B, 7A), and 9 SNPs that were unmapped (Table 4). Additionally, mapping of resistance was carried out for a new virulent race TKTTF of Ethiopian origin. A genome-wide scan for resistance loci resulted in 109 SNPs distributed on five chromosomes (1A, 4A, 5A, 6B, 7A), and 2 unmapped SNPs (Table 5).

**Table 4:** List of SNPs significantly associated with seedling resistance to race TRTTF

SNP	Chr^a^	Pos (cM)	*P* value	R^2^ (%)^b^
IWB843	0	0.0	1.43E-04	15.4
IWB8113			8.36E-04	13.2
IWB9699			8.36E-04	12.9
IWB25143			4.62E-07	10.8
IWB27028			5.95E-04	10.7
IWB48121			1.83E-04	7.7
IWB64530			8.36E-04	7.6
IWB67724			2.26E-04	6.1
IWB68822			8.36E-04	4.2
IWB9794	1B	43.9	6.06E-04	4.2
IWB72495		53.3	6.06E-04	4.1
IWB11356		62.4	5.66E-04	4.0
IWB11357			5.66E-04	3.4
IWB60559			6.06E-04	3.2
IWB65404			4.44E-04	3.2
IWB11819		62.6	6.06E-04	3.2
IWB11820			4.44E-04	3.2
IWB34561			6.06E-04	3.6
IWB47684			7.00E-04	3.6
IWB53143			9.38E-04	3.5
IWB6592			6.06E-04	3.5
IWB41306		64.9	6.06E-04	3.4
IWB44021	1D	8.7	5.95E-04	3.4
IWA8551		50.6	7.02E-04	3.4
IWB24961	5D	200.3	3.70E-04	3.4
IWB57210			3.70E-04	3.4
SNP	Chr^a^	Pos (cM)	P value	R^2^ (%)^b^
IWB12448	6A	1.9	4.82E-04	3.4
IWB33595		3.4	1.14E-04	3.4
IWB11274		4.7	3.93E-08	3.3
IWB53755			1.25E-04	3.2
IWA5416		5.6	2.81E-09	3.4
IWA5781		5.7	3.17E-09	3.3
IWB7601		6.4	1.85E-05	3.7
IWB47842		7.0	1.31E-04	3.7
IWA3856		12.5	1.85E-05	28.6
IWA6871			1.85E-05	27.2
IWB23520			1.85E-05	25.7
IWB2392			6.41E-12	25.7
IWB26415			1.85E-05	24.7
IWB43804			1.85E-05	24.7
IWB9075			3.00E-06	24.6
IWA7006		12.8	2.06E-05	24.3
IWB22036		12.9	4.64E-20	20.2
IWB10105		13.5	5.80E-10	16.4
IWB11315			7.37E-18	15.4
IWB23521			5.98E-13	15.4
IWB26414			6.11E-12	15.4
IWB35219			1.18E-17	15.4
IWB43805			1.98E-18	14.8
IWB43810			1.85E-05	14.8
IWB58271			2.78E-19	14.8
IWB60233			6.11E-12	14.8
IWB6358			1.85E-05	12.5
IWB66015			6.11E-12	12.5
IWB67413			7.37E-18	12.5
SNP	Chr^a^	Pos (cM)	P value	R^2^ (%)^b^
IWB67415	6A		8.35E-18	12.4
IWB72957			1.98E-18	12.4
IWA272		15.7	1.50E-04	12.4
IWB64917			3.29E-04	11.9
IWB5029		16.0	2.84E-15	11.8
IWB35595		16.6	2.57E-12	10.0
IWB43808			2.57E-12	9.1
IWB64918			2.57E-12	7.6
IWB72956			2.57E-12	6.5
IWA7913		17.0	2.57E-12	6.1
IWB23519			5.44E-10	6.0
IWA705		20.0	5.56E-06	6.0
IWA4962		21.1	4.26E-07	5.4
IWB31479			4.72E-07	5.4
IWB48751			1.01E-08	5.4
IWB20143		22.0	2.44E-10	5.4
IWA4551		22.9	6.94E-11	5.4
IWA4552			1.11E-10	5.4
IWB22191			6.67E-06	5.4
IWB28421			2.44E-10	5.4
IWB50019			2.44E-10	5.3
IWB28338		23.0	6.67E-06	4.8
IWB1550		25.5	2.19E-10	4.4
IWB22216			5.42E-05	4.3
IWB30507			2.19E-10	4.3
IWB40111			5.36E-06	3.8
IWB52325			2.06E-10	3.5
IWB64084.2	6B	9.8	3.68E-05	5.3
IWB11653.2		14.5	2.10E-05	5.0
SNP	Chr^a^	Pos (cM)	P value	R^2^ (%)^b^
IWB14901	7A	124.3	2.72E-04	3.9
IWB48466		217.0	8.24E-04	3.2

^a^Chr ‘0’ indicates unmapped SNPs that were significant in the analysis

^b^R^2^Indicates the percent of phenotypic variation explained by the significant locus

**Table 5:** List of SNPs significantly associated with seedling resistance to race TKTTF

SNP	Chr^a^	Pos (cM)	P value	R^2^ (%)^b^
IWB31876	0	0.0	8.08E-04	9.7
IWB71333			4.84E-04	9.6
IWB57448	1A	21.5	8.57E-04	9.3
IWA8622		24.4	2.26E-04	9.2
IWB22324	4A	142.3	5.22E-06	9.1
IWA4651		144.4	3.10E-08	8.8
IWB27971			1.97E-05	7.5
IWB34478			3.37E-05	6.8
IWB34733			1.54E-11	4.9
IWB3569			3.95E-05	3.9
IWB61312			3.95E-05	3.7
IWB63979			1.19E-09	3.5
IWB68386			8.24E-04	3.2
IWA1505		145.2	1.15E-08	3.1
IWA3449		147.1	3.83E-05	3.9
IWB2554			1.61E-05	3.2
IWB62397			3.09E-04	13.9
IWB12146		150.7	5.47E-07	11.1
IWB47019			4.39E-07	9.2
IWB59346			5.93E-06	8.7
IWB1407		151.2	5.19E-05	8.1
IWB14910			3.47E-05	8.0
IWB71978			3.47E-05	8.0
IWB59368		151.3	3.14E-04	7.8
IWB68322			9.43E-06	7.6
IWB53393		153.0	1.35E-04	7.6
SNP	Chr^a^	Pos (cM)	P value	R^2^ (%)^b^
IWB35434	4A	154.1	8.01E-04	7.5
IWB51926		154.3	2.22E-04	7.5
IWB59099			9.09E-06	7.4
IWB70193			5.34E-06	7.4
IWB35545		163.8	9.37E-07	7.4
IWA1410		164.1	5.23E-08	7.1
IWA4084			2.39E-08	7.0
IWA4858			4.55E-07	6.9
IWA5353			1.49E-06	6.9
IWA7364			2.46E-08	6.5
IWA7365			1.38E-08	6.4
IWB21715			5.74E-08	6.0
IWB23332			2.85E-07	6.0
IWB26256			2.37E-06	6.0
IWB26495			1.85E-07	5.7
IWB275			3.45E-07	5.7
IWB27679			5.06E-04	5.4
IWB29568			1.69E-07	5.4
IWB3001			3.44E-07	5.3
IWB31447			5.51E-07	5.0
IWB34609			1.17E-06	4.9
IWB36388			4.52E-07	4.9
IWB4517			1.46E-05	4.9
IWB48829			9.10E-07	4.9
IWB49256			5.08E-07	4.7
IWB52393			1.16E-06	4.2
IWB6097			2.58E-06	3.9
IWB72383			2.60E-08	3.7
IWB9276			1.90E-07	3.7
SNP	Chr^a^	Pos (cM)	P value	R^2^ (%)^b^
IWA2224	5A	88.0	8.03E-04	3.5
IWA2836		94.9	5.08E-04	3.2
IWB34927			9.63E-04	3.2
IWB72540	6B	108.9	6.96E-04	3.5
IWA3268		109.9	2.33E-04	3.2
IWA5605			3.19E-04	3.1
IWA5606			9.46E-04	10.1
IWB56595			2.40E-04	9.3
IWB2749		110.4	7.59E-04	9.3
IWB2751			4.59E-04	8.3
IWB43467			5.88E-04	7.4
IWB48603			4.34E-04	7.3
IWB50367			1.37E-04	6.7
IWB56594			5.88E-04	6.7
IWB61565			3.01E-04	6.7
IWB65679			6.45E-04	6.1
IWB43133		113.3	5.16E-04	5.3
IWB61528			5.16E-04	5.2
IWB14375		113.7	1.68E-04	4.8
IWB1747			5.46E-04	4.7
IWB30381			7.14E-04	4.4
IWB41515			4.78E-05	4.2
IWB57727			7.14E-04	4.2
IWB58200			3.42E-04	4.2
IWB59006			1.88E-05	4.2
IWB59306			6.29E-07	4.1
IWB70316			2.35E-04	4.1
IWB72471			1.52E-04	4.1
IWB9416			4.59E-06	4.1
SNP	Chr^a^	Pos (cM)	P value	R^2^ (%)^b^
IWA4245	6B	114.4	2.10E-04	4.0
IWA4246		116.2	8.75E-04	3.9
IWB28557			4.91E-04	3.9
IWB59175.2		119.0	4.47E-04	3.9
IWB24880		120.3	1.35E-04	3.8
IWB24881			8.13E-04	3.7
IWB41216			7.51E-04	3.7
IWB24882		120.6	1.35E-04	3.5
IWB3553			5.79E-05	3.5
IWB45581			2.20E-05	3.5
IWB46893			9.30E-05	3.5
IWB66027			5.14E-07	3.4
IWB10711.2		121.8	2.38E-08	3.4
IWB23602			1.39E-04	3.4
IWB23603			1.86E-04	3.4
IWB40587			1.69E-04	3.4
IWB44802			1.57E-06	3.3
IWB73072			2.25E-08	3.3
IWB48548		121.9	1.57E-06	3.3
IWB28880		122.1	1.57E-06	3.3
IWB44669			1.08E-07	3.3
IWB464			7.18E-04	3.3
IWB71190		122.2	2.71E-04	3.2
IWB43213		122.3	1.69E-04	3.2
IWB41217		122.9	6.96E-04	3.2
IWB47075			6.28E-09	3.2
IWB34899.2	7A	6.4	3.22E-05	5.0

^a^Chr ‘0’ indicates unmapped SNPs that were significant in the analysis

^b^R^2^Indicates the percent of phenotypic variation explained by the significant locus

## Discussion

Wheat stem rust disease has been primarily controlled by the use of resistant genes discovered in hexaploid wheat and its related species. However, the Ug99 race group has defeated many of the widely deployed resistance genes, and thus poses a threat to wheat production globally. Moreover, several of the previously identified genes discovered in wild progenitors or landraces are not desirable for their use in resistance breeding because of linkage drag [3, 44]. Therefore, discovery of loci contributing resistance to Ug99 and other virulent races in elite breeding germplasm is a clear advantage. The resistance uncovered in this study, composed of elite germplasm from North American breeding programs, can provide a great resource for the fight against Ug99 and stem rust in general. As no SNP markers were significant across all four field environments, differences among the disease environments with regard to races present, temperature, and other environmental factors as well as locus by environment interaction are likely involved in this lack of consistency. Lack of strong correlations among the environments also corroborates this assumption (Table 1).

### Comparison of significant APR Loci with published studies

The map locations of significant SNP markers in our study, obtained from Wang *et al*. [39], were compared to positions of markers and genes/quantitative trait loci (QTL) reported in previous mapping studies conducted to uncover loci associated with stem rust resistance. In this section, we have used from the integrated genetic map consisting of different marker types generated by Maccaferri *et al*. [45] to obtain the relative distances between previously reported markers and the significant markers in our study.

Five significant SNP markers (*IWA3120*, *IWB21176*, *IWB31027*, *IWB56771*, *IWB59663*) were detected at position 90 cM on chromosome 1B, of which all except *IWA3120* were also detected in Ethiopia 2013. The marker *cfd48* reported by Pozniak *et al*. [46] in a durum wheat (*Triticum durum* Desf.) GWAS study is located 4 cM from the SNP markers we detected, and could represent the same locus. Bhavani *et al*. [47] and Njau *et al*. [48] both reported the marker *wPt-1560* on 1BL to be associated with Ug99 resistance in separate spring wheat RIL populations. This marker, as well as *Sr58*, an APR gene for stem rust of wheat [49, 50], are located at a distance of >50 cM from these five SNP markers. QTL on chromosome 2A providing APR to Ug99 have also been mainly reported in durum wheat mapping populations. Letta *et al*. [51] detected *gwm1045* to be significantly associated with Ug99 resistance in a durum wheat AM panel; and Haile *et al*. [52] reported a QTL linked to the marker *gwm1198* on 2A that confers resistance to Ug99 in the durum wheat population Kristal/Sebatel. Neither of these markers was in proximity to the SNP markers detected in our study. We detected one significant marker, *IWB8481*, located at 9 cM on chromosome 2D. The only reported QTL on 2D that provides APR to Ug99 and its derivative races is in the CIMMYT biparental population PBW343/Kiritati [47]. Two *Sr* genes – *Sr32*, and *Sr46* have been mapped to the short arm of 2D [49, 53], and both provide resistance to Ug99 [44]. It should be noted that *Sr32* has also been introgressed to 2A and 2B [54], but is not expected to be present in the 250 lines analyzed in this study. We used the *Sr32* markers developed by Mago *et al.* [53] to screen our panel but found the markers to be not predictive of the gene (Additional file 1). As no reliable marker for *Sr46* has been developed, we are unable to distinguish between these two genes and the marker we found on 2D.

The marker *IWA4275* detected on chromosome 2B (position 197 cM) in our study is very close (distance of 2.7 cM) to the marker *wPt-8460*, known to be linked to *Sr9h* in 1956 Rockefeller Foundation cultivar Gabo 56 (CI 14035) [11]. The same marker was also reported by Yu *et al*. [55] in their association mapping study constituting of CIMMYT spring wheat germplasm. *Sr9h*, previously temporarily designated as *SrWeb*, is derived from the Canadian wheat cultivar ‘Webster’ (RL6201) and confers ASR gene effective to TTKSK [56]. Markers developed by Rouse *et al*. [11] showed that *Sr9h* is present in 13 lines (5 %) in our panel (Additional file 1), implying that *IWA4275* could represent the *Sr9h* locus in our panel. The gene *Sr9a* is also located on 2BL [55, 57], but is ineffective to Ug99 [44].

On 2B, we also detected five SNP markers: *IWA8534*, *IWB23660*, *IWB25868*, *IWB69631*, and *IWB25869* located at 126 cM. Based on the consensus map published by Maccaferri *et al*. [45], these markers are located at a distance of 4 cM from *wmc332*, which is linked to *Sr28* [58]. We used two markers: *wmc332* [58] and a newly developed SNP marker (Michael Pumphrey, personal communication) to investigate if *Sr28* was present in our panel. While both markers have only been tested on a limited panel of lines and are not confirmed as diagnostic, we detected that up to 20 lines (8 %) could possess *Sr28* (Additional file 1). Thus, our marker-trait association may be detecting *Sr28* on this 2B region.

Nine SNP markers were detected on the short arm of chromosome 3B with 8 SNPs located at positional range of 11.5 – 14.1 cM and one additional SNP at 32.2 cM. These 8 SNPs in the range 11.5 – 14.1 cM may be proximal to *Sr2*, a highly important APR gene for stem rust of wheat [3, 59]. Upon marker screening, it was found that 22 lines (9 %) in the panel contain *Sr2* (Additional file 1). This gene is used extensively in the CIMMYT spring wheat breeding program, and is shared by some US breeding programs that also incorporated this gene in their germplasm for broad-spectrum resistance. It is possible that the SNP at 32.2 cM is associated with Ug99 resistance that has been observed near the *Sr12* locus [60]. Another stem rust APR gene, *Sr57* [61], is located on chromosome 7D. Screening of the panel using the sequence-tagged site marker developed by Lagudah *et al*. [62] showed that 97 lines (39 %) could contain *Sr57*. For other two stem rust APR genes: *Sr55*, located on 4D [63] and *Sr56*, located on 5B [64], no diagnostic markers are available. As no SNP markers were detected on chromosomes 4D and 5B during the analysis, we believe these genes are not present in our mapping panel.

Several QTL located on chromosome 4A that provide resistance to Ug99 have been reported in association mapping studies [55, 65, 66], and in biparental studies [47] in CIMMYT germplasm. These sources of stem rust resistance are not located in the vicinity of the SNPs *IWB46973*, *IWB56556*, and *IWB67877* detected also on 4A in our study. Similarly, QTL on chromosome 5A providing resistance against Ug99 have been reported in biparental and association mapping studies [46, 47]. However, chromosome positions of the QTL and significant loci reported in these studies differ from those detected in our study.

We detected only one significant SNP (*IWA233*) on 6AS. Mapped at 66 cM, this SNP is located away (>100 cM) from the marker *gwm617* reported by Pozniak *et al*. [46], and from the marker *Sr26#43* linked to *Sr26*, which provides resistance to the Ug99 and its derivative races [55, 67]. Marker screening confirmed that *Sr26* is absent in the panel under study (Additional file 1). Several QTL effective to Ug99 and its derivative races have also been discovered on chromosome 6B [4, 49]. Of the reported QTL, the DArT marker *wPt-6116* in the AM study conducted by Yu *et al*. [65] is located very close to the significant markers detected in this study: 1.1 cM from *IWB24757* and 2.2 cM from *IWB45581*. The gene *Sr11* is located on 6BL, but is ineffective to Ug99 and its derivative races [3, 5]. Likewise, several QTL have been reported on 7A that provide field resistance to Ug99 [46, 47, 51, 52, 68]. However, none of the reported QTL or positions of significant marker effects coincide with the significant markers detected in this study. Two 7B SNP markers – *IWB47548* and *IWA4175* – were significantly associated with resistance to Ug99. Letta *et al*. [51] have reported loci associated with resistance to Ug99 in durum wheat germplasm, however they are located at a large distance (>50 cM) from both markers in our study.

The significant SNP markers associated with APR to Ug99 reported in this study provide several resistance loci to fight the disease, of which some are likely novel. Validation of the significant markers in all chromosomes is essential to confirm the identity of the associated resistance loci as well as to test their usefulness in marker assisted resistance breeding in breeding programs.

### Comparison of significant seedling-resistance Loci with existing resistance genes

The results of the GWAS for seedling-resistance in this study were compared with previous findings for ASR to stem rust of wheat. As the discovered SNPs are suspected to be linked primarily with existing or putatively novel resistance genes, a search for similarities in chromosomal location with known resistance genes was emphasized.

We detected 23 SNPs in our germplasm panel that were significantly associated with race TTKSK resistance at the seedling stage. The SNP marker *IWA642* mapped at 67.7 cM on 1D is relatively close to *Sr50*, a gene that provides resistance to the Ug99 group of races [28]. The seedling resistance genes *SrCad* and *SrTmp* are considered to be present in the panel used in this study. *SrCad* is a stem rust resistance gene derived from the Canadian wheat lines ‘Peace’ and ‘AC Cadillac’, and is effective to Ug99 and its derivative races [69]. Located on chromosome 6D, this gene confers a highly resistant reaction (IT of 1 to 12) to TTKSK in seedling stages, and is moderately resistant to Ug99 in field nurseries. *SrCad* has not been shown to be different than *Sr42* in either map position or resistance specificity [70]. *SrTmp* is another gene resistant to TTKSK yet no SNPs were detected on 4B where the *SrTmp* gene is thought to be located [3]. Additional data suggest that *SrTmp* may be located on 6DS at a similar location to *Sr42*/*SrCad* [71]. We used two markers: SSR marker *cfd49* [72] and a SNP marker (Gao *et al.*, unpublished) to screen the panel for presence/absence of *Sr42*. Results indicated that at least 71 or more lines in the panel could carry this gene, yet the markers did not support each other (Additional file 1). Neither marker results also corroborate our TTKSK seedling screening results. We are not aware of any study carried out on broad germplasm to determine if these two markers are diagnostic or even predictive. From our results, it appears that they are neither diagnostic nor predictive of *Sr42*/*SrCad*. Similarly, *Sr9h* has a resistant reaction to race TTKSK at the seedling stages (1 to 2 infection type) [56]. The presence of *Sr9h* was confirmed by marker screening, as discussed above. Except for the likely presence of *Sr9h*, the position of the loci conferring Ug99 resistance in this study suggest that different genes than the ones discussed above could be present in our panel. We suspect that association mapping is limited by the low frequency of resistance loci (only 15 (6 %) of 250 lines resistant to TTKSK), leading to lack of detection of SNPs significantly associated with *SrCad* or *SrTmp*. Since no ASR genes effective to Ug99 are known to exist on chromosomes 3B and 5B, our findings indicate that the North American breeding germplasm might contain previously undiscovered important sources of resistance to the disease.

The genes *Sr24* and *Sr36* are resistant to the race TTKSK, yet no SNPs associated with resistance to this race were detected in the chromosomes containing these genes. *Sr24*, located on 3DL, is widely used in Mexico and the USA; *Sr36*, located on 2BS, is known to be present in wheat lines in the USA [3]. Lack of detection of these genes can be attributed to either 1) representative germplasm with these genes are not present in our GWAS panel; or 2) if present, the allele frequency is very low which does not pass our stringent analysis filters. Upon marker screening (Additional file 1), we discovered that *Sr36* is not present in our panel; and only 7 lines (3 %) contain *Sr24*, confirming our assumptions.

Of the 77 mapped SNPs significantly associated with resistance to TRTTF, 57 SNPs were located on the short arm of chromosome 6A (position range 2 cM – 26 cM). These markers are most likely linked to the gene *Sr8a* which is located on 6AS and is effective to the race TRTTF [34, 73]. Similarly, the SNP marker *IWB48466* located on the long arm of 7A (217 cM) is in the same region as the stem rust resistance gene *Sr22*. This gene was introgressed into 7AL of hexaploid wheat from its diploid relative *Triticum boeoticum* [74], and is effective against TRTTF [75]. Marker screening of the GWAS panel with a robust sequence tagged site (STS) marker developed by Periyannan *et al*. [76] confirmed that *Sr22* is not present in the panel. *Sr31*, while ineffective against TTKSK, is effective against TRTTF, and is located on 1BL [75, 77]. Our GWAS results detected 13 significant SNPs, all on the short arm of chromosome 1B (position range 44 cM – 65 cM). Given the presence of CIMMYT lines in our panel and the widespread use of *Sr31* in breeding programs, screening of lines with *Sr31* with these markers is needed to determine if the markers are linked to *Sr31*, or if a novel source of resistance to TRTTF is located on 1BS.

Chromosome 1DS is known to harbor multiple *Sr* genes [49], and could be represented by the two SNPs that were detected on 1DS in our analysis. We also discovered markers on 5DL and 6BS associated with resistance to TRTTF. As no ASR genes effective against TRTTF are known to exist on 5DL and 6BS, the North American elite breeding germplasm likely possesses novel genes for resistance to the Yemeni stem rust race TRTTF.

One-hundred and nine SNPs associated with seedling resistance to the newly detected Ethiopian stem rust race TKTTF were detected on five chromosomes: 1AS, 4AL, 5AL, 6BL, and 7AS. The 52 6BL SNPs distributed in the positional range of 109 cM – 123 cM likely represent the gene *Sr11* which is effective to this race. Fifty-one significant SNPs were located on 4AL (142 cM – 164 cM) possibly indicative of resistance gene *Sr7a* (TKTTF is virulent to *Sr7b*). No ASR genes are known to be located on chromosome 5AL, and therefore the germplasm under study may possess a new source of resistance to the race TKTTF in this region. APR QTL providing resistance to the Ug99 and its derivative races have been detected in the 1AS region [49, 55]. Additionally, the gene *Sr1RS*^*Amigo*^ is located on the 1RS.1AL rye chromosome arm translocation. Chromosome 7AS does not possess any known ASR genes, yet APR QTL effective to Ug99 and its derivative races have been detected in the region [49, 68].

None of the SNPs associated with seedling resistance for the three races were common, suggesting that none of the genes in this material are broadly effective. Further studies involving development of populations for fine mapping and allelism tests are required to elaborate and confirm the nature of the genetic mechanisms controlling the resistance to these three rust races.

### Using breeding lines in GWAS

One of the main advantages of conducting association mapping on a panel consisting of breeding germplasm is to explore the genetic composition of the lines, and estimate the effects of significantly associated loci with the trait(s) of interest. The discovery of significant SNPs can allow for tagging of lines that are enriched for alleles associated with the trait, and their use in gene introgression for resistance breeding. More importantly, as the lines used in this AM study are elite, they possess the desired agronomic traits, and are adapted to the desired regions. This helps in avoiding the problems that could otherwise arise from linkage drag, when more diverse germplasm is used to introgress alleles of interest. Singh *et al*. [5] have reported that up to 95 % of germplasm from global seed collections and breeding programs are susceptible to Ug99. As Ug99 and its derivative races have not yet been observed in North America, it is prudent to prepare for their possible arrival by developing resistant varieties. Discovery of resistant sources in existing breeding programs can speed up the process of gene introgression into elite lines, gene pyramiding for elevated resistance to the disease, and possible identification of diagnostic markers that can be used in marker assisted resistance breeding. Germplasm sharing among the breeding programs for this purpose, at least within the US, is plausible given the genetic similarity among the lines, as observed in Fig. 2. The availability of SNP alleles associated with reduced disease severity (as well as increased severity) in both adult plant and seedling stages (Additional file 1) should be useful for breeders to make decisions about selection of lines to be used as parents in their breeding programs. Breeders may also use the significant SNP markers we have provided to design assays for possible marker assisted selection or screening of resistant materials in their own breeding programs. Additionally, Table 6 has been populated with a list of lines that exhibited high levels of APR to Ug99 (Table 6), and seedling resistance to TTKSK (Table 7). The complete genotypic and phenotypic data presented in this study have also been made available on The Triticeae Toolbox (T3 webportal) with the goal of facilitating line selection based on *Sr* marker associations. We are confident that the North American wheat breeding programs can fortify the stem rust resistance in their germplasm by capitalizing on the information provided in this study.

**Table 6:** Elite spring wheat lines from North American breeding programs that exhibit high level of adult plant resistance (APR) to Ug99 in four field environments

Line^a^	Origin^b^	Environment^c^				Avg Severity^d^
Park	Alberta	KenOff13	KenMain13	EthOff13	EthOff14	18
		25	5	20	20	
9262	CIMMYT	NA	5	30	30	22
AC_Cadillac	Manitoba	18	5	20	2	11
AC_Splendor	Manitoba	8	5	11	NA	8
Glencross	Manitoba	18	15	40	5	19
Peace	Manitoba	NA	5	10	5	7
Fortuna	MSU	8	5	6	10	7
Hi-Line	MSU	NA	5	30	30	22
Newana	MSU	NA	5	13	20	13
Thatcher	MSU	10	5	10	20	11
AC_Eatonia	Saskatchewan	NA	5	40	10	18
CDC_Alsak	Saskatchewan	5	5	35	10	14
CDC_Osler	Saskatchewan	NA	5	5	5	5
Neepawa	Saskatchewan	8	5	35	10	14
Roblin	Saskatchewan	NA	5	25	NA	15
Selkirk	Saskatchewan	10	5	10	50	19
10010-20	UCD	15	5	20	10	13
UC1642	UCD	NA	5	45	20	23
UC1682	UCD	10	5	25	50	23
MN03119-4	UMN	NA	10	45	20	25
MN03148	UMN	25	5	25	20	19
MN08013-2	UMN	10	5	30	50	24
Line^a^	Origin^b^	Environment^c^				Avg Severity^d^
HW080169	WSU	NA	5	40	30	25
Avg_APR_Lines	-	18	8	31	28	21
Avg_GWAS_Panel	-	33	35	52	50	43

^a^‘Avg_APR_Lines’ represents the mean disease severity (%) across the lines showing high level of APR, and ‘Avg_GWAS_Panel’ represents the mean disease severity among all lines in the GWAS panel

^b^Source (breeding program) of the line showing APR to Ug99

^c^Disease severity (%) for each environment

^d^The average disease severity (%) across four environments

**Table 7:** Elite spring wheat lines from North American breeding programs that exhibit high level of seedling resistance to race TTKSK. For each line, the observed seedling infection type (IT) for each race and the corresponding value on the linear scale are presented under the column ‘IT’ and ‘Linear Score’, respectively

Line^a^	Origin^b^	TTKSK		TRTTF		TKTTF	
9253	CIMMYT	IT	Linear Score	IT	Linear Score	IT	Linear Score
		2- / 3+	4	2 / 3+	5	2-	4
9262	CIMMYT	2	5	2	5	;2-	1
9263	CIMMYT	;12-	1	;1	1	0;/33+	0
AC_Cadillac	Manitoba	22+	5	2	5	0;1	1
Peace	Manitoba	22+	5	2-;	3	0;	0
Hi-Line	MSU	0;3-	2	2-	4	33-	9
MT0415	MSU	2	5	33+	8	01	1
Thatcher	MSU	0; / 3+	0	33+	8	33+	8
AC_Crystal	Saskatchewan	2+	6	2	5	32+	7
AC_Karma	Saskatchewan	2	5	2	5	22+	5
AC_Vista	Saskatchewan	22+	5	2	5	22+	5
SD4214	SDSU	2+	6	3+	9	12-	3
SD4279	SDSU	2	5	2	5	33+	9
PI610750	UCD	22+	5	2-	4	2-	4
UC1643	UCD	2	5	22-	5	0;1	1
Avg_Resistant_Lines	-	-	4	-	5	-	4
Avg_GWAS_Panel	-	-	8	-	7	-	4

^a^‘Avg_ Resistant_Lines’ and ‘Avg_GWAS_Panel’ represent the mean linear score among lines resistant to TTKSK, and all lines in the GWAS panel, respectively

^b^Source (breeding program) of the line showing APR to Ug99

## Conclusions

In this study, we report the frequency and variability in seedling resistance and APR present in North American spring wheat breeding germplasm to virulent exotic *Pgt* races. Several loci were found to be significant, which is an indication that despite the relatively narrow goals for germplasm development, enough genetic variation lies within the current North American breeding germplasm that can be utilized to breed for resistance against the virulent stem rust races, including the Ug99 race group. While only a small portion (6 %) of the germplasm showed seedling resistance, APR to Ug99 revealed several likely-novel genomic regions associated with resistance to Ug99. The lines that performed well at either or both growth stages (seedling and adult) could be used immediately to make crosses with elite lines to generate lines with improved rust resistance. Specific crosses could also be made to create mapping populations to fine map the regions of interest in an effort to identify diagnostic markers linked with the resistance loci. The discovery of such diagnostic markers will add great value to recurrent selection breeding programs as well as in identification of lines that carry the resistance loci. As such, further characterization and validation of the detected loci is necessary for effective utilization of these results. The availability of marker and trait data on the T3 webportal that were generated for this GWAS panel should enable interested groups to pursue these studies.

## Availability of supporting data

The genotypic data generated on this GWAS panel and used in this article are available under the genotyping experiment ‘TCAP90K_SpringAM_panel’ in *The Triticeae Toolbox repository,*https://triticeaetoolbox.org/wheat/display_genotype.php?trial_code=TCAP90K_SpringAM_panel*).* The phenotypic data collected on this GWAS panel and used in this article are available *under the experiment set ‘USSpring_GWAS’* in *The Triticeae Toolbox repository,*https://triticeaetoolbox.org/wheat/view.php?table=experiment_set&uid=48*).* The data sets supporting the results of this article are included within the article and its additional files.

## Additional file

Additional file 1:
**Additional details on data analysis on the GWAS panel.** The file should be opened in Microsoft Office Excel. (XLSX 433 kb)

## Abbreviations

*Pgt*: *Puccinia graminis* f. sp. *tritici*
ASR: All-stage resistance
APR: Adult plant resistance
QTL: Quantitative trait locus
AM: Association mapping
LD: Linkage disequilibrium
GWAS: Genome-wide association study
PCA: Principal component analysis.
