# Erratum to: Association mapping of North American spring wheat breeding germplasm reveals loci conferring resistance to Ug99 and other African stem rust races

**DOI:** 10.1186/s12870-015-0684-1

**Published:** 2016-01-19

**Authors:** P. Bajgain, M. N. Rouse, P. Bulli, S. Bhavani, T. Gordon, R. Wanyera, P. N. Njau, W. Legesse, J. A. Anderson, M. O. Pumphrey

**Affiliations:** Department of Agronomy, Purdue University, 915 West State Street, West Lafayette, 47907 IN USA; Department of Agronomy and Plant Genetics, University of Minnesota, St. Paul, 55108 MN USA; United States Department of Agriculture-Agricultural Research Service (USDA-ARS), Cereal Disease Laboratory, St. Paul, 55108 MN USA; Department of Plant Pathology, University of Minnesota, St. Paul, 55108 MN USA; Department of Crop and Soil Sciences, Washington State University, Pullman, 99164 WA USA; International Maize and Wheat Improvement Center (CIMMYT), ICRAF House, United Nations Avenue, Gigiri, Nairobi Kenya; United States Department of Agriculture-Agricultural Research Service (USDA-ARS), Aberdeen, 83210 ID USA; Kenya Agricultural and Livestock Research Organization (KALRO), Njoro, Kenya; Ethiopian Institute of Agricultural Research (EIAR), Pawe, Ethiopia

## Erratum

Upon publication of this article [1] it was noticed that Tables Two-Seven (Tables 1, 2, 3, 4, 5 and 6 here) were formatted incorrectly. The Tables have now been updated in the original article and can also be seen below.

**Table 1:** List of SNPs significantly associated with APR to the Ug99 race group in Kenya and Ethiopia. ‘Chromosome 0’ includes all unmapped SNP markers

SNP^a^	Chr^b^	Pos (cM)	*P* value	R^2^ (%)^c^	KenOff13^d^	KenMain13^e^	EthOff13^f^	EthOff14^g^
IWB1351	0	0.0	9.18E-04	1.4	-	-	-	+
**IWB11987**	0	0.0	2.71E-04	4.0	-	-	-	-
IWB13304	0	0.0	9.18E-04	1.4	-	-	-	+
IWB20617	0	0.0	8.92E-04	0.5	-	-	-	-
IWB40153	0	0.0	9.18E-04	1.4	-	-	-	+
**IWB65634**	0	0.0	2.18E-04	3.9	-	-	-	-
IWA3120	1B	90.3	9.07E-04	0.6	-	-	-	-
IWB21176	1B	90.3	9.64E-04	0.6	-	-	+	-
IWB31027	1B	90.3	9.64E-04	0.6	-	-	+	-
IWB56771	1B	90.3	9.34E-04	0.6	-	-	+	-
IWB59663	1B	90.3	9.64E-04	0.6	-	-	+	-
IWB49915	2A	122.5	9.18E-04	1.4	-	-	-	+
IWB49914	2A	123.6	9.18E-04	1.4	-	-	-	+
IWB22672	2A	159.7	9.98E-04	0.6	-	-	+	-
IWB2369	2B	48.5	7.68E-04	0.4	-	-	-	-
IWA4275	2B	105.9	8.75E-04	1.4	-	-	-	-
IWA8534	2B	126.1	1.80E-04	0.3	-	+	-	-
IWB23660	2B	126.3	1.79E-04	0.3	-	+	-	-
IWB25868	2B	126.3	3.11E-04	0.7	-	+	-	-
IWB69631	2B	126.3	3.11E-04	0.7	-	+	-	-
IWB25869	2B	126.5	3.11E-04	0.7	-	+	-	-
IWB32143	2B	157.2	5.05E-04	4.6	-	-	-	-
IWB8481	2D	9.2	8.44E-04	0.5	-	-	-	-
IWA5203	3B	11.5	7.97E-04	0.4	-	-	-	-
IWB30730	3B	11.5	7.74E-04	1.3	-	-	-	-
**IWB12193**	3B	11.6	3.24E-04	4.2	-	+	-	-
IWB49924	3B	11.6	5.65E-04	0.2	-	-	-	-
IWB65737	3B	11.6	9.15E-04	0.6	-	-	-	-
IWB60424	3B	13.8	5.02E-04	0.1	-	-	-	-
IWB36021	3B	14.1	8.31E-04	1.3	-	-	-	-
IWA2493	3B	32.2	1.49E-04	0.2	-	-	+	-
IWB40004	4A	30.9	9.12E-04	0.6	-	+	-	-
**IWB52694**	4A	43.4	9.96E-05	3.2	-	+	-	-
IWB46973	4A	47.0	8.26E-04	1.3	-	+	-	-
IWB56556	4A	47.0	7.54E-04	1.3	-	-	-	-
IWB67877	4A	47.0	8.34E-04	1.3	-	-	-	-
IWB47184	5A	69.6	3.44E-04	0.3	-	-	+	-
IWA233	6A	66.0	8.48E-04	1.3	-	+	-	-
IWB24757	6B	119.7	6.33E-04	1.1	-	-	-	-
IWB35697	6B	119.7	1.77E-05	1.3	-	-	+	+
IWB6474	6B	119.7	6.33E-04	1.1	-	-	-	-
IWB45581	6B	120.6	3.00E-04	0.6	-	-	-	-
**IWB5070**	7A	211.0	2.19E-04	3.9	-	-	-	-
IWB1874	7A	212.7	3.18E-04	0.3	-	-	-	-
**IWB4830**	7A	212.7	4.50E-04	4.5	-	-	+	-
IWB62560	7A	213.2	2.30E-04	0.6	-	-	-	-
IWB47548	7B	153.4	6.50E-04	0.3	-	-	-	-
IWA4175	7B	177.1	9.52E-04	0.6	-	-	-	-

^a^Underlined SNP markers were detected during the ‘combined’ mapping approach but not during the ‘APR-specific’ mapping approach. SNP markers in bold were detected in both mapping approaches

^b^Chr ‘0’ indicates unmapped SNPs that were significant in the analysis

^c^R^2^ Indicates percent of phenotypic variation explained by the significant locus

^d-g^The ‘+’ sign indicates that the SNP was also detected in GWAS results in each of the environments. The ‘-’ sign indicates that the SNP was detected only in combined analysis of all environments, and not in individual environments

**Table 2:** List of SNPs significantly associated with seedling resistance to Ug99 (race TTKSK)

SNP	Chr^a^	Pos (cM)	*P* value	R^2^ (%)^b^
IWB2159	0	0.0	3.15E-06	4.8
IWB14222	0	0.0	3.45E-04	2.8
IWB36324	0	0.0	6.44E-04	2.5
IWB37066	0	0.0	3.62E-06	4.8
IWB40312	0	0.0	1.97E-07	6.1
IWB49537	0	0.0	2.81E-04	2.9
IWB68926	0	0.0	3.96E-05	3.7
IWA642	1D	67.7	1.32E-04	3.2
IWB24497	3B	67.5	8.43E-06	4.4
IWB30621	3B	67.5	1.97E-07	6.1
IWB42046	3B	67.5	8.27E-08	6.5
IWB4823	3B	67.5	8.43E-06	4.4
IWB56471	3B	67.5	1.97E-07	6.1
IWB61425	3B	67.5	8.43E-06	4.4
IWB59929	3B	74.4	3.91E-05	3.7
IWB9451	3B	76.9	3.36E-04	2.8
IWA5363	4A	40.3	7.90E-04	2.4
IWA3394	5B	132.3	2.29E-05	3.9
IWB7593	5B	132.3	6.14E-04	2.5
IWB822	5B	134.1	5.30E-04	2.6
IWB46318	5B	215.7	1.56E-05	4.1
IWA2099	5B	216.7	9.72E-05	3.3
IWA2100	5B	216.7	1.13E-04	3.3

^a^Chr ‘0’ indicates unmapped SNPs that were significant in the analysis

^b^R^2^ Indicates the percent of phenotypic variation explained by the significant locus

**Table 3:** List of SNPs significantly associated with seedling resistance to race TRTTF

SNP	Chr^a^	Pos (cM)	*P* value	R^2^ (%)^b^
IWB843	0	0.0	1.43E-04	15.4
IWB8113	0	0.0	8.36E-04	13.2
IWB9699	0	0.0	8.36E-04	12.9
IWB25143	0	0.0	4.62E-07	10.8
IWB27028	0	0.0	5.95E-04	10.7
IWB48121	0	0.0	1.83E-04	7.7
IWB64530	0	0.0	8.36E-04	7.6
IWB67724	0	0.0	2.26E-04	6.1
IWB68822	0	0.0	8.36E-04	4.2
IWB9794	1B	43.9	6.06E-04	4.2
IWB72495	1B	53.3	6.06E-04	4.1
IWB11356	1B	62.4	5.66E-04	4.0
IWB11357	1B	62.4	5.66E-04	3.4
IWB60559	1B	62.4	6.06E-04	3.2
IWB65404	1B	62.4	4.44E-04	3.2
IWB11819	1B	62.6	6.06E-04	3.2
IWB11820	1B	62.6	4.44E-04	3.2
IWB34561	1B	62.6	6.06E-04	3.6
IWB47684	1B	62.6	7.00E-04	3.6
IWB53143	1B	62.6	9.38E-04	3.5
IWB6592	1B	62.6	6.06E-04	3.5
IWB41306	1B	64.9	6.06E-04	3.4
IWB44021	1D	8.7	5.95E-04	3.4
IWA8551	1D	50.6	7.02E-04	3.4
IWB24961	5D	200.3	3.70E-04	3.4
IWB57210	5D	200.3	3.70E-04	3.4
IWB12448	6A	1.9	4.82E-04	3.4
IWB33595	6A	3.4	1.14E-04	3.4
IWB11274	6A	4.7	3.93E-08	3.3
IWB53755	6A	4.7	1.25E-04	3.2
IWA5416	6A	5.6	2.81E-09	3.4
IWA5781	6A	5.7	3.17E-09	3.3
IWB7601	6A	6.4	1.85E-05	3.7
IWB47842	6A	7.0	1.31E-04	3.7
IWA3856	6A	12.5	1.85E-05	28.6
IWA6871	6A	12.5	1.85E-05	27.2
IWB23520	6A	12.5	1.85E-05	25.7
IWB2392	6A	12.5	6.41E-12	25.7
IWB26415	6A	12.5	1.85E-05	24.7
IWB43804	6A	12.5	1.85E-05	24.7
IWB9075	6A	12.5	3.00E-06	24.6
IWA7006	6A	12.8	2.06E-05	24.3
IWB22036	6A	12.9	4.64E-20	20.2
IWB10105	6A	13.5	5.80E-10	16.4
IWB11315	6A	13.5	7.37E-18	15.4
IWB23521	6A	13.5	5.98E-13	15.4
IWB26414	6A	13.5	6.11E-12	15.4
IWB35219	6A	13.5	1.18E-17	15.4
IWB43805	6A	13.5	1.98E-18	14.8
IWB43810	6A	13.5	1.85E-05	14.8
IWB58271	6A	13.5	2.78E-19	14.8
IWB60233	6A	13.5	6.11E-12	14.8
IWB6358	6A	13.5	1.85E-05	12.5
IWB66015	6A	13.5	6.11E-12	12.5
IWB67413	6A	13.5	7.37E-18	12.5
IWB67415	6A	13.5	8.35E-18	12.4
IWB72957	6A	13.5	1.98E-18	12.4
IWA272	6A	15.7	1.50E-04	12.4
IWB64917	6A	15.7	3.29E-04	11.9
IWB5029	6A	16.0	2.84E-15	11.8
IWB35595	6A	16.6	2.57E-12	10.0
IWB43808	6A	16.6	2.57E-12	9.1
IWB64918	6A	16.6	2.57E-12	7.6
IWB72956	6A	16.6	2.57E-12	6.5
IWA7913	6A	17.0	2.57E-12	6.1
IWB23519	6A	17.0	5.44E-10	6.0
IWA705	6A	20.0	5.56E-06	6.0
IWA4962	6A	21.1	4.26E-07	5.4
IWB31479	6A	21.1	4.72E-07	5.4
IWB48751	6A	21.1	1.01E-08	5.4
IWB20143	6A	22.0	2.44E-10	5.4
IWA4551	6A	22.9	6.94E-11	5.4
IWA4552	6A	22.9	1.11E-10	5.4
IWB22191	6A	22.9	6.67E-06	5.4
IWB28421	6A	22.9	2.44E-10	5.4
IWB50019	6A	22.9	2.44E-10	5.3
IWB28338	6A	23.0	6.67E-06	4.8
IWB1550	6A	25.5	2.19E-10	4.4
IWB22216	6A	25.5	5.42E-05	4.3
IWB30507	6A	25.5	2.19E-10	4.3
IWB40111	6A	25.5	5.36E-06	3.8
IWB52325	6A	25.5	2.06E-10	3.5
IWB64084.2	6B	9.8	3.68E-05	5.3
IWB11653.2	6B	14.5	2.10E-05	5.0
IWB14901	7A	124.3	2.72E-04	3.9
IWB48466	7A	217.0	8.24E-04	3.2

^a^Chr ‘0’ indicates unmapped SNPs that were significant in the analysis

^b^R^2^ Indicates the percent of phenotypic variation explained by the significant locus

**Table 4:** List of SNPs significantly associated with seedling resistance to race TKTTF

SNP	Chr^a^	Pos (cM)	*P* value	R^2^ (%)^b^
IWB31876	0	0.0	8.08E-04	9.7
IWB71333	0	0.0	4.84E-04	9.6
IWB57448	1A	21.5	8.57E-04	9.3
IWA8622	1A	24.4	2.26E-04	9.2
IWB22324	4A	142.3	5.22E-06	9.1
IWA4651	4A	144.4	3.10E-08	8.8
IWB27971	4A	144.4	1.97E-05	7.5
IWB34478	4A	144.4	3.37E-05	6.8
IWB34733	4A	144.4	1.54E-11	4.9
IWB3569	4A	144.4	3.95E-05	3.9
IWB61312	4A	144.4	3.95E-05	3.7
IWB63979	4A	144.4	1.19E-09	3.5
IWB68386	4A	144.4	8.24E-04	3.2
IWA1505	4A	145.2	1.15E-08	3.1
IWA3449	4A	147.1	3.83E-05	3.9
IWB2554	4A	147.1	1.61E-05	3.2
IWB62397	4A	147.1	3.09E-04	13.9
IWB12146	4A	150.7	5.47E-07	11.1
IWB47019	4A	150.7	4.39E-07	9.2
IWB59346	4A	150.7	5.93E-06	8.7
IWB1407	4A	151.2	5.19E-05	8.1
IWB14910	4A	151.2	3.47E-05	8.0
IWB71978	4A	151.2	3.47E-05	8.0
IWB59368	4A	151.3	3.14E-04	7.8
IWB68322	4A	151.3	9.43E-06	7.6
IWB53393	4A	153.0	1.35E-04	7.6
IWB35434	4A	154.1	8.01E-04	7.5
IWB51926	4A	154.3	2.22E-04	7.5
IWB59099	4A	154.3	9.09E-06	7.4
IWB70193	4A	154.3	5.34E-06	7.4
IWB35545	4A	163.8	9.37E-07	7.4
IWA1410	4A	164.1	5.23E-08	7.1
IWA4084	4A	164.1	2.39E-08	7.0
IWA4858	4A	164.1	4.55E-07	6.9
IWA5353	4A	164.1	1.49E-06	6.9
IWA7364	4A	164.1	2.46E-08	6.5
IWA7365	4A	164.1	1.38E-08	6.4
IWB21715	4A	164.1	5.74E-08	6.0
IWB23332	4A	164.1	2.85E-07	6.0
IWB26256	4A	164.1	2.37E-06	6.0
IWB26495	4A	164.1	1.85E-07	5.7
IWB275	4A	164.1	3.45E-07	5.7
IWB27679	4A	164.1	5.06E-04	5.4
IWB29568	4A	164.1	1.69E-07	5.4
IWB3001	4A	164.1	3.44E-07	5.3
IWB31447	4A	164.1	5.51E-07	5.0
IWB34609	4A	164.1	1.17E-06	4.9
IWB36388	4A	164.1	4.52E-07	4.9
IWB4517	4A	164.1	1.46E-05	4.9
IWB48829	4A	164.1	9.10E-07	4.9
IWB49256	4A	164.1	5.08E-07	4.7
IWB52393	4A	164.1	1.16E-06	4.2
IWB6097	4A	164.1	2.58E-06	3.9
IWB72383	4A	164.1	2.60E-08	3.7
IWB9276	4A	164.1	1.90E-07	3.7
IWA2224	5A	88.0	8.03E-04	3.5
IWA2836	5A	94.9	5.08E-04	3.2
IWB34927	5A	94.9	9.63E-04	3.2
IWB72540	6B	108.9	6.96E-04	3.5
IWA3268	6B	109.9	2.33E-04	3.2
IWA5605	6B	109.9	3.19E-04	3.1
IWA5606	6B	109.9	9.46E-04	10.1
IWB56595	6B	109.9	2.40E-04	9.3
IWB2749	6B	110.4	7.59E-04	9.3
IWB2751	6B	110.4	4.59E-04	8.3
IWB43467	6B	110.4	5.88E-04	7.4
IWB48603	6B	110.4	4.34E-04	7.3
IWB50367	6B	110.4	1.37E-04	6.7
IWB56594	6B	110.4	5.88E-04	6.7
IWB61565	6B	110.4	3.01E-04	6.7
IWB65679	6B	110.4	6.45E-04	6.1
IWB43133	6B	113.3	5.16E-04	5.3
IWB61528	6B	113.3	5.16E-04	5.2
IWB14375	6B	113.7	1.68E-04	4.8
IWB1747	6B	113.7	5.46E-04	4.7
IWB30381	6B	113.7	7.14E-04	4.4
IWB41515	6B	113.7	4.78E-05	4.2
IWB57727	6B	113.7	7.14E-04	4.2
IWB58200	6B	113.7	3.42E-04	4.2
IWB59006	6B	113.7	1.88E-05	4.2
IWB59306	6B	113.7	6.29E-07	4.1
IWB70316	6B	113.7	2.35E-04	4.1
IWB72471	6B	113.7	1.52E-04	4.1
IWB9416	6B	113.7	4.59E-06	4.1
IWA4245	6B	114.4	2.10E-04	4.0
IWA4246	6B	116.2	8.75E-04	3.9
IWB28557	6B	116.2	4.91E-04	3.9
IWB59175.2	6B	119.0	4.47E-04	3.9
IWB24880	6B	120.3	1.35E-04	3.8
IWB24881	6B	120.3	8.13E-04	3.7
IWB41216	6B	120.3	7.51E-04	3.7
IWB24882	6B	120.6	1.35E-04	3.5
IWB3553	6B	120.6	5.79E-05	3.5
IWB45581	6B	120.6	2.20E-05	3.5
IWB46893	6B	120.6	9.30E-05	3.5
IWB66027	6B	120.6	5.14E-07	3.4
IWB10711.2	6B	121.8	2.38E-08	3.4
IWB23602	6B	121.8	1.39E-04	3.4
IWB23603	6B	121.8	1.86E-04	3.4
IWB40587	6B	121.8	1.69E-04	3.4
IWB44802	6B	121.8	1.57E-06	3.3
IWB73072	6B	121.8	2.25E-08	3.3
IWB48548	6B	121.9	1.57E-06	3.3
IWB28880	6B	122.1	1.57E-06	3.3
IWB44669	6B	122.1	1.08E-07	3.3
IWB464	6B	122.1	7.18E-04	3.3
IWB71190	6B	122.2	2.71E-04	3.2
IWB43213	6B	122.3	1.69E-04	3.2
IWB41217	6B	122.9	6.96E-04	3.2
IWB47075	6B	122.9	6.28E-09	3.2
IWB34899.2	7A	6.4	3.22E-05	5.0

^a^Chr ‘0’ indicates unmapped SNPs that were significant in the analysis

^b^R^2^ Indicates the percent of phenotypic variation explained by the significant locus

**Table 5:** Elite spring wheat lines from North American breeding programs that exhibit high level of adult plant resistance (APR) to Ug99 in four field environments

Line^a^	Origin^b^	Environment^c^				Avg severity^d^
		KenOff13	KenMain13	EthOff13	EthOff14	
Park	Alberta	25	5	20	20	18
9262	CIMMYT	NA	5	30	30	22
AC_Cadillac	Manitoba	18	5	20	2	11
AC_Splendor	Manitoba	8	5	11	NA	8
Glencross	Manitoba	18	15	40	5	19
Peace	Manitoba	NA	5	10	5	7
Fortuna	MSU	8	5	6	10	7
Hi-Line	MSU	NA	5	30	30	22
Newana	MSU	NA	5	13	20	13
Thatcher	MSU	10	5	10	20	11
AC_Eatonia	Saskatchewan	NA	5	40	10	18
CDC_Alsak	Saskatchewan	5	5	35	10	14
CDC_Osler	Saskatchewan	NA	5	5	5	5
Neepawa	Saskatchewan	8	5	35	10	14
Roblin	Saskatchewan	NA	5	25	NA	15
Selkirk	Saskatchewan	10	5	10	50	19
10010-20	UCD	15	5	20	10	13
UC1642	UCD	NA	5	45	20	23
UC1682	UCD	10	5	25	50	23
MN03119-4	UMN	NA	10	45	20	25
MN03148	UMN	25	5	25	20	19
MN08013-2	UMN	10	5	30	50	24
HW080169	WSU	NA	5	40	30	25
Avg_APR_Lines	-	18	8	31	28	21
Avg_GWAS_Panel	-	33	35	52	50	43

^a^‘Avg_APR_Lines’ represents the mean disease severity (%) across the lines showing high level of APR, and ‘Avg_GWAS_Panel’ represents the mean disease severity among all lines in the GWAS panel

^b^Source (breeding program) of the line showing APR to Ug99

^c^Disease severity (%) for each environment

^d^The average disease severity (%) across four environments

**Table 6:** Elite spring wheat lines from North American breeding programs that exhibit high level of seedling resistance to race TTKSK. For each line, the observed seedling infection type (IT) for each race and the corresponding value on the linear scale are presented under the column ‘IT’ and ‘Linear Score’, respectively

Line^a^	Origin^b^	TTKSK		TRTTF		TKTTF	
		IT	Linear Score	IT	Linear Score	IT	Linear Score
9253	CIMMYT	2- / 3+	4	2 / 3+	5	2-	4
9262	CIMMYT	2	5	2	5	;2-	1
9263	CIMMYT	;12-	1	;1	1	0; / 33+	0
AC_Cadillac	Manitoba	22+	5	2	5	0;1	1
Peace	Manitoba	22+	5	2-;	3	0;	0
Hi-Line	MSU	0;3-	2	2-	4	33-	9
MT0415	MSU	2	5	33+	8	01	1
Thatcher	MSU	0; / 3+	0	33+	8	33+	8
AC_Crystal	Saskatchewan	2+	6	2	5	32+	7
AC_Karma	Saskatchewan	2	5	2	5	22+	5
AC_Vista	Saskatchewan	22+	5	2	5	22+	5
SD4214	SDSU	2+	6	3+	9	12-	3
SD4279	SDSU	2	5	2	5	33+	9
PI610750	UCD	22+	5	2-	4	2-	4
UC1643	UCD	2	5	22-	5	0;1	1
Avg_Resistant_Lines	-	-	4	-	5	-	4
Avg_GWAS_Panel	-	-	8	-	7	-	4

^a^‘Avg_ Resistant_Lines’ and ‘Avg_GWAS_Panel’ represent the mean linear score among lines resistant to TTKSK, and all lines in the GWAS panel, respectively

^b^Source (breeding program) of the line showing APR to Ug99
